# Study on the death and disease burden caused by high sugar-sweetened beverages intake in China from 1990 to 2019

**DOI:** 10.1093/eurpub/ckac067

**Published:** 2022-07-21

**Authors:** Yingying Jiang, Tingling Xu, Wenlan Dong, Cordia Chu, Maigeng Zhou

**Affiliations:** National Center for Chronic and Noncommunicable Disease Control and Prevention, Chinese Center for Disease Control and Prevention, Beijing, China; Centre for Environment and Population Health, School of Medicine, Griffith University, Brisbane, Queensland, Australia; National Center for Chronic and Noncommunicable Disease Control and Prevention, Chinese Center for Disease Control and Prevention, Beijing, China; National Center for Chronic and Noncommunicable Disease Control and Prevention, Chinese Center for Disease Control and Prevention, Beijing, China; Centre for Environment and Population Health, School of Medicine, Griffith University, Brisbane, Queensland, Australia; National Center for Chronic and Noncommunicable Disease Control and Prevention, Chinese Center for Disease Control and Prevention, Beijing, China

## Abstract

**Background:**

To analysis the death and disease burden caused by high sugar-sweetened beverages intake in China from 1990 to 2019.

**Methods:**

Data were obtained from the 2019 Global Burden of Disease Study. We analyzed the death cases caused by high sugar-sweetened beverages intake in China and provinces from 1990 to 2019, as well as the disease burden (including disability-adjusted of life year), years of life lost and years lived with disability, and compared the changes of death in 1990 and 2019.

**Results:**

In 2019, the number of deaths attributed to sugar-sweetened beverages in China reached 46 633 with an increase of 95% compared with 1990. The proportion of deaths caused by excessive consumption of carbon-containing beverages increased from 0.34% in 1990 to 0.46% in 2019, an increase of 35%. In 2019, the top five provinces in China with more deaths caused by excessive intake of sugary beverages were Shandong, Henan, Hebei, Hunan and Guangdong, with the number of death cases 4337, 3881, 3010, 2762 and 2611, respectively.

**Conclusions:**

The number of deaths and disease burdens caused by high sugar-sweetened beverages intake in China has increased significantly over the past three decades. The burden of disease due to high intake of sugary beverages varies widely from province to province. We suggest that attention should be paid to the problem of excessive intake of high sugar-sweetened beverages for Chinese population. In addition to regular monitoring and investigation of sugar-sweetened beverage intake, comprehensive measures should be taken in China’s sugar control work.

## Introduction

Sugar-sweetened beverages refer to beverages added with any form of sugar (such as brown sugar, corn sugar, corn syrup, fructose, glucose, etc.). Common sugary drinks include soft drinks, fruit juices, sports drinks, energy and vitamin drinks, coffee and tea with added sugar.^1^ The ‘Dietary Guidelines for Chinese Residents (2016)’ defines sugar-sweetened beverages as beverages that are artificially added with sugar and have a sugar content of more than 5% in the process of making beverages.^2^ Current studies have confirmed that taking an extra sugar-sweetened beverage per day increases the risk of hypertension by 8%, the risk of coronary heart disease by 17%,^3^ and the risk of type 2 diabetes by 30%.^4^ Excessive intake of sugary drinks can also increase the risk of obesity, kidney disease, non-alcoholic liver disease, dental caries and gout.^5^^,^^6^

The intake of sugar-sweetened beverages is as addictive behavior as smoking and drinking and is an integral part of the lifestyle and eating habits of residents in modern society. The global consumption of sugar-sweetened beverages has grown rapidly, and the per capita intake of soft drinks has increased from 9.5 l in 1997 to 11.4 l in 2010. As an important part of sugary beverages, carbonated beverages have been continuously increasing in global sales. The consumption of sugar-sweetened beverages by residents of developing countries such as China has increased significantly.^7^^,^^8^ Research by Guo Haijun and others found that half of Chinese adults consume sugary beverages, among which carbonated beverages are the most common type.^7^ From 2003 to 2014, the per capita consumption of beverages in China has increased significantly. In 2014, the per capita consumption of beverages was as high as 119 kg, which was approximately 10 times the per capita consumption (12 kg) in 2003.^9^

The ‘Dietary Guidelines for Chinese Residents (2016)’ pointed out that the intake of added sugars should be limited with a daily intake under exceed 50 g and it is best to be controlled below 25 g.^2^ In July 2019, the State Council of China launched the ‘Healthy China Action (2019-2030)’. In the special action on reasonable diet, one of the 15 special actions, whether the daily intake of oil, salt and sugar exceeds the standard is used as specific indicators for assessment of the actions. These documents have clarified the specific tasks of reducing salt, oil and sugar with health promotion measures.^10^ However, compared with the epidemiological studies and intervention practices for oil, salt and other chronic disease-related risk factors, the epidemiology study of sugar intake is limited in China. Therefore, this study will analyze the overall and provincial deaths and disease burdens attributed to the excessive intake of sugar-sweetened beverages in the Chinese population, aiming to provide science for the targeted research and development of sugar control policies and the development of sugar control interventions.

## Methods

### Data source

The data required for this study are all from Global Burden of Disease (GBD) 2019 of China. GBD 2019 uses a unified and comparable method to comprehensively analyze and estimate the prevalence (rate) and disease burden of 369 diseases or injuries in 204 countries and regions from 1990 to 2019. The burden of disease attributable to 87 risk factors has been systematically sorted out. The background and methods of this research are in the literature.^11–13^ This study selected death data and disease burden data attributed to excessive intake of sugar-sweetened beverages for analysis. Among them, disease burden includes disability-adjusted of life year (DALYs), years of life lost (YLLs) and years lived with disability (YLDs). DALYs = YLLs + YLDs, the estimation methods of YLLs and YLDs are detailed in the literature.^11^^,^^14^^,^^15^ The GBD 2019 China Study uses the global burden of disease method to systematically analyze all available demographic and epidemiological data at the provincial level in China (excluding Taiwan, China).

### Principles of attributable disease burden

*Population attributable fraction (PAF)*: The PAF attributable to high-sugar beverages is calculated according to the theory of comparative risk. The calculation method and formula of PAF are detailed in the literature.^16^ The data used for the attribution analysis of the disease burden caused by the intake of sugar-sweetened beverages in the Chinese population comes from Euromonitor International. Sugar-sweetened beverage exposure refers to daily intake of ≥50 kcal/226.8 g carbonated drinks, soda water, energy drinks, fruit juices, but excluding 100% pure fruit juice and vegetable juice.^11^ The minimum theoretical exposure distribution of sugar-sweetened beverages is 0–5 g/day, which means that the risk of disease or death in the population is considered to be the lowest in this distribution. The current GBD calculation process has re-evaluated all existing evidence of causality between sugar-sweetened beverages and disease outcomes, and found sufficient evidence for the causal relationship between sugar-sweetened beverages and ischemic heart disease (IHD) as well as type 2 diabetes. Therefore, the current calculations have updated the method and quantified the burden of disease caused by the direct impact of sugar-sweetened beverages on disease outcomes.^11^

*Calculation of the burden of disease attributable to sugar-sweetened beverages*: The number of deaths from various related death diseases attributed to sugar-sweetened beverages = PAF × M, where M is the total deaths of a disease that is etiologically related to sugar-sweetened beverages. Age-standardized populations in the GBD were calculated by using the GBD world population age standard. The non-weighted mean of 2019 age-specific proportional distributions from the GBD 2019 population estimates for all national locations with a population greater than 5 million people in 2019 to generate an updated standard population age structure.

## Results

### Deaths attributed to the consumption of high-sugar beverages in the Chinese population

In 1990, the number of deaths attributed to sugar-sweetened beverages in China was 23 974. The number of deaths is increasing over time. In 2019, the number of deaths attributed to sugar-sweetened beverages in China reached 46 633 with an increase of 95%. During the same period, the standardized mortality rate attributable to sugary beverages dropped from 3.86 per 100 000 to 2.94 per 100 000, with a decrease of 24%. The proportion of deaths caused by excessive consumption of carbon-containing beverages increased from 0.34% in 1990 to 0.46% in 2019, an increase of 35%. Compared with 1990, the number of deaths attributed to high intake of sugar-sweetened beverages by men in China increased by 100% in 2019, which was higher than the increase in the proportion of female deaths (87%). There were 26 826 deaths and 19 807 women in 2019. During the same period, the increase in the proportion of deaths attributed to excessive intake of sugary drinks by women (39%) was higher than that of men (35%). With the increase of age, the growth rate of the number of deaths caused by excessive intake of sugary beverages is increasing, and the growth rate of the number of deaths caused by sugary beverages for people 15–49, 50–69 and ≥70 years old are 1%, 17% and 163%, respectively. Compared with 1990, the proportion of deaths attributed to excessive intake of sugar-sweetened beverages in the 15- to 49-year-old Chinese population increased with the greatest extent of 43%. See table 1 for details.

**Table 1:** Comparison of deaths attributed to sugar-sweetened beverages among Chinese population (95% UI)

	1990			2019			Changes (1990 vs. 2019)		
	Number	Rate (/100 000)	PAF (%)	Number	Rate (/100 000)	PAF (%)	Number	Rate	PAF
Male	13 381.09	4.86	0.37	26 826.50	4.16	0.49	1.00	−0.14	0.35
	(10 156.05–7069.12)	(3.65–6.23)	(0.28–0.46)	(18 351.05–36 127.64)	(2.81–5.64)	(0.35–0.67)	(0.35–1.95)	(−0.49 to 0.30)	(−0.07 to 0.90)
Female	10 593.66	3.19	0.33	19 807.08	2.19	0.45	0.87	−0.31	0.39
	(7570.56–13 847.45)	(2.27–4.25)	(0.24–0.42)	(13 034.84–28 191.27)	(1.41–3.16)	(0.30–0.63)	(0.16–1.85)	(−0.64 to 0.21)	(−0.08 to 0.99)
15–49 year-old^a^	2187.67	0.33	0.18	2220.45	0.31	0.26	0.01	−0.06	0.43
	(1539.67–2892.98)	(0.23–0.43)	(0.13–0.23)	(1108.13–3343.86)	(0.15–0.46)	(0.14–0.36)	(−0.47 to 0.61)	(−0.53 to 0.79)	(−0.22 to 1.12)
50–69 year-old^a^	8879.10	5.76	0.37	10 414.98	2.82	0.35	0.17	−0.51	−0.05
	(6565.083–11 406.82)	(4.26–7.40)	(0.28–0.46)	(6870.56–14 136.66)	(1.86–3.83)	(0.24–0.44)	(−0.26 to 0.74)	(−0.75 to −0.09)	(−0.37 to 0.32)
≥70 year-old^a^	12 907.98	33.74	0.40	33 998.14	31.49	0.51	1.63	−0.07	0.28
	(9666.46–16 246.89)	(25.27–2.47)	(0.30–0.50)	(24 183.18–45 346.62)	(22.40–42.00)	(0.37–0.67)	(0.88–2.69)	(−0.34 to 0.31)	(−0.09 to 0.77)
Total	23 974.75	3.86	0.34	46 633.57	2.94	0.46	0.95	−0.24	0.35
	(19 260.28–29 126.38)	(3.02–4.75)	(0.27–0.41)	(34 038.47–61 191.67)	(2.10–3.89)	(0.34–0.60)	(0.43–1.56)	(−0.50 to 0.07)	(0.00–0.73)

a The death rate of each age group is crude death rate.

### Chronic disease deaths and disease burden caused by high-sugar beverages

The most common chronic disease deaths caused by sugary drinks are IHD and diabetes mellitus. From 1990 to 2019, the number of deaths from IHD and that of diabetes increased by 95% and 94%, respectively. The number of deaths in 2019 was 42 098 for IHD and 4534 deaths for diabetes. Compared with the measurement in 1990, the DALYs of IHD and the DALYs of diabetes attributed to high intake of sugary beverages in 2019 increased by 49% and 88%, respectively, and increased to 757 416 and 258 774, respectively in 2019. Compared with YLLs, the increase in YLDs caused by excessive supplementation of sugar-sweetened beverages is more significant, especially the increase of YLDs of diabetes due to excessive intake of sugar-sweetened beverages is 105%. See table 2 for details.

**Table 2:** Chronic disease deaths and disease burden attributable to sugar-sweetened beverages in the Chinese population (95% UI)

	IHD						DM					
	1990		2019		Change (1990 vs. 2019)		1990		2019		Changes (1990 vs. 2019)	
	Number	Rate (/100 000)	Number	Rate (/100 000)	Number	Rate	Number	Rate (/100 000)	Number	Rate (/100 000)	Number	Rate
Death	21 639.80	3.54	42 098.91	2.69	0.95	−0.24	2334.95	0.32	4534.66	0.25	0.94	−0.21
	(17 308.84–26 273.08)	(2.76–4.38)	(30 310.5–56 071.22)	(1.92–3.59)	(0.41–1.58)	(−0.46 to 0.01)	(1850.66–2890.48)	(0.25–0.39)	(2949.70–5970.44)	(0.17–0.33)	(0.31–1.63)	(−0.46 to 0.06)
DALYs	510 000.80	64.76	75 7416.50	41.78	0.49	−0.35	137 769.90	15.59	258 774.10	12.89	0.88	−0.17
	(406 904.03–25 564.93)	(52.2–78.76)	(52 7185.52–00 4008.57)	(29.74–55.36)	(0.06–0.98)	(−0.54 to −0.15)	(102 896.74–81 392.85)	(11.74–20.42)	(15 3961.95–367 693.94)	(7.72–18.33)	(0.23–1.48)	(−0.45 to 0.08)
YLLs	485 009.30	61.55	71 8441.00	39.74	0.48	−0.35	55 020.21	6.48	88 824.77	4.48	0.61	−0.31
	(385 330.95–593 974.90)	(49.52–74.62)	(49 9226.65–95 7741.04)	(28.05–52.73)	(0.06–0.99)	(−0.54 to −0.14)	(44 097.93–68 039.24)	(5.18–8.00)	(56 052.79–117 955.76)	(2.86–5.93)	(0.07–1.22)	(−0.53 to −0.06)
YLDs	24 991.47	3.21	38 975.49	2.03	0.56	−0.37	82 749.68	9.11	169 949.30	8.41	1.05	−0.08
	(15 933.62–36 765.54)	(2.06–4.69)	(22 416.75–60 306.25)	(1.17–3.12)	(0.11–0.98)	(−0.55 to −0.20)	(54 034.81–12 0221.16)	(6.00–13.18)	(93 454.58–265 523.98)	(4.63–13.06)	(0.33–1.68)	(−0.40 to 0.21)

### Disease burden caused by high-sugar beverages

The DALYs of the burden of disease for death in the Chinese population caused by excessive intake of sugary beverages increased from 647 770.70 in 1990 to 1 016 191.00 in 2019, an increase of 57%, and the PAF also reached 26% (0.20% in 1990 and 0.25% in 2019). Over the same period, the standardized mortality rate for death caused by excessive intake of sugary beverages dropped by 32%. The increase in the number of YLLs and YLDs caused by excessive intake of sugar-sweetened beverages reached 49% and 94%, respectively. PAF of YLLs increased by 54%, and PAF of YLDs decreased by 10%. See table 3 for details.

**Table 3:** Disease burden of the Chinese population attributable to excessive intake of sugary beverages (95% UI)

		1990	2019	Changes (1990 vs. 2019)
DALYs	Number	647 770.70 (523 495.27–87 242.30)	1 016 191.00 (734 173.20–1 302 281.98)	0.57 (0.15 to 1.03)
	Rate (/100 000)	80.35 (65.52–97.08)	54.67 (40.05–70.13)	−0.32 (−0.50 to 0.13)
	PAF (%)	0.20 (0.16–0.23)	0.25 (0.18–0.31)	0.26 (−0.03 to 0.58)
YLLs	Number	540 029.50 (432 763.37–58 687.29)	807 265.80 (571 674.81–1 058 512.27)	0.49 (0.08 to 0.98)
	Rate(/100 000)	68.03 (55.12–82.26)	44.23 (31.87–57.72)	−0.35 (−0.53 to 0.14)
	PAF (%)	0.22 (0.18–0.26%)	0.33 (0.25–0.41)	0.54 (0.16 to 0.97)
YLDs	Number	107 741.16 (70 817.96–151 454.24)	208 924.84 (123 053.00–312 130.23)	0.94 (0.36 to 1.47)
	Rate(/100 000)	12.33 (8.20–17.32)	10.44 (6.10–15.55)	−0.15 (−0.41 to 0.09)
	PAF (%)	0.13 (0.10–0.16)	0.12 (0.08–0.15)	−0.10 (−0.37 to 0.16)

### Deaths attributed to sugary drinks in various provinces

In 2019, the top five provinces in China with more deaths caused by excessive intake of sugary beverages were Shandong, Henan, Hebei, Hunan and Guangdong, with the number of death cases 4337, 3881, 3010, 2762 and 2611, respectively. A fewer deaths occurred in Macau, Tibet and Qinghai in 2019. Compared with 1990, the top five provinces with the fastest increase in deaths caused by excessive intake of sugary beverages in 2019 were Chongqing, Qinghai, Gansu, Xinjiang and Hunan, with increases of 241%, 160%, 156%, 155% and 140%, respectively. Compared with 1990, Henan, Qinghai, and Gansu displayed an increase in the standardized mortality rate attributable to excessive intake of sugary beverages, while the remaining provinces showed negative growth. During the same period, the proportion of deaths caused by excessive intake of sugary beverages in Hunan, Gansu, Shandong, Henan and Yunnan increased significantly, with an increase of more than 60% (66% in Hunan, 64% in Gansu, 64% in Shandong and 61% in Henan, and Yunnan 61%). The PAF of Hong Kong Special Administrative Region fell by 15%. See table 4 for details.

**Table 4:** The burden of deaths attributed to sugar-sweetened beverages among populations in provinces in China

Location	1990			2019			Changes (1990 vs. 2019)		
	Number	Rate(/100 000)	PAF (%)	Number	Rate(/100 000)	PAF (%)	Number	Rate	PAF
Anhui	963.63	3.46	0.30	1941.98	2.68	0.43	1.02	−0.23	0.45
Beijing	336.00	4.95	0.55	686.02	2.40	0.59	1.04	−0.52	0.07
Chongqing	248.46	2.88	0.23	848.05	2.43	0.34	2.41	−0.16	0.47
Fujian	419.55	2.88	0.26	783.86	1.92	0.36	0.87	−0.33	0.39
Gansu	329.90	3.39	0.28	846.17	3.41	0.46	1.56	0.01	0.64
Guangdong	1180.15	3.41	0.32	2611.27	2.38	0.51	1.21	−0.30	0.56
Guangxi	742.51	3.30	0.30	1531.46	2.99	0.41	1.06	−0.09	0.40
Guizhou	494.59	3.12	0.22	763.29	2.21	0.27	0.54	−0.29	0.24
Hainan	124.47	3.63	0.34	268.28	2.98	0.44	1.16	−0.18	0.27
Hebei	1439.27	3.98	0.38	3010.33	3.81	0.51	1.09	−0.04	0.33
Heilongjiang	1090.37	8.12	0.64	2256.76	5.60	0.73	1.07	−0.31	0.15
Henan	1819.43	3.80	0.37	3881.00	3.92	0.60	1.13	0.03	0.61
HK SAR	105.55	2.28	0.39	194.08	1.14	0.33	0.84	−0.50	−0.15
Hubei	1032.52	3.65	0.32	1619.29	2.54	0.39	0.57	−0.30	0.21
Hunan	1148.72	3.50	0.30	2762.43	3.43	0.49	1.40	−0.02	0.66
Inner Mongolia	532.46	5.88	0.48	1032.77	4.21	0.59	0.94	−0.28	0.22
Jiangsu	1077.85	2.62	0.27	1742.53	1.45	0.30	0.62	−0.45	0.10
Jiangxi	764.65	4.03	0.29	1121.67	2.52	0.39	0.47	−0.37	0.34
Jilin	890.16	7.84	0.59	1488.24	4.83	0.71	0.67	−0.38	0.20
Liaoning	1351.00	6.77	0.65	2605.45	4.48	0.68	0.93	−0.34	0.03
Macao SAR	10.92	4.22	0.67	20.36	2.25	0.68	0.86	−0.47	0.01
Ningxia	91.55	5.44	0.44	208.51	4.13	0.55	1.28	−0.24	0.26
Qinghai	59.99	3.73	0.26	156.03	3.76	0.39	1.60	0.01	0.47
Shaanxi	735.14	4.55	0.36	1488.90	3.88	0.55	1.03	−0.15	0.55
Shandong	1890.44	3.82	0.37	4377.09	3.53	0.60	1.32	−0.08	0.64
Shanghai	351.99	3.33	0.39	694.06	1.73	0.43	0.97	−0.48	0.09
Shanxi	711.67	4.73	0.41	1395.30	3.90	0.54	0.96	−0.18	0.33
Sichuan	2117.19	3.65	0.30	2500.77	2.50	0.33	0.18	−0.32	0.09
Tianjin	282.63	5.38	0.57	575.10	3.48	0.64	1.03	−0.35	0.11
Tibet	46.87	4.08	0.21	52.53	2.63	0.25	0.12	−0.36	0.18
Xinjiang	361.64	5.69	0.37	922.63	5.05	0.52	1.55	−0.11	0.40
Yunnan	571.33	3.04	0.22	1273.08	2.97	0.35	1.23	−0.02	0.61
Zhejiang	652.15	2.43	0.25	974.29	1.24	0.27	0.49	−0.49	0.07
Total	23 974.75	3.86	0.34	46 633.57	2.94	0.46	0.95	−0.24	0.35

## Discussion

In 2019, the number of deaths attributed to excessive intake of sugar-sweetened beverages in China was 46 633. Compared with 1990, the number of deaths increased by 95%. The global average increase was 30.4%.^11^ Over the same period, the increase in PAF of deaths caused by excessive intake of sugary beverages also reached 35%. These results suggest that in the past 30 years, the death threats faced by the Chinese population from excessive intake of sugary beverages have increased. The number of deaths attributed to sugar-sweetened beverages among Chinese men in 2019 and their increase in the past three decades are higher than those of women. The possible reason is that the death rate of body mass index related diseases in Chinese men has been consistently higher than that of women.^17^^,^^18^ Excessive intake of sugar-sweetened beverages, as a behavioral risk factor, is also the same as high body mass index, high blood pressure, red meat intake, alcohol drinking and smoking, all of which are becoming popular with the development of society and economy.^11^ In the 1970s, the dietary patterns of people in European and American countries changed, which was reflected in the increase in the proportion of processed food intake (including sugary drinks) and the increase in the frequency of eating out. In recent years, with economic and social development as well as urbanization, the dietary patterns of residents in developing countries have also undergone similar changes, and the changes have become more rapid. Chinese traditional dietary habits have gradually changed into reduced cereal intake, meat and food consumption. The intake of oil and high sugar has increased rapidly.^19^ In recent years, the sales of carbonated beverages represented by Coca-Cola and Pepsi in China has increased by more than 100%. In the same period, the USA, as a traditional carbonated beverage consumer, has slightly reduced its consumption. The declining market share of beverage sales shows that China and other regions with rapid economic growth in recent years have become the focus of the marketing of sugary beverage commercial groups.^20^ Studies by Chinese scholars have shown that in recent years, the frequency of consumption of sugary drinks by Chinese residents has increased significantly, and the energy supply ratio of sugary drinks in the dietary structure has also increased.^7^^,^^8^

Zhou *et al.*^18^ has categorized China into five types of regions with different health characteristics based on the life expectancy and death patterns in different provinces of China from 1990 to 2013. In this study, the increase in standardized mortality due to excessive intake of sugar-sweetened beverages in Henan, Qinghai and Gansu provinces, which is to a certain extent in line with the results of Zeng’s research. Zeng’s findings showed that high body mass index causes a faster increase in standardized mortality in the provinces which overlapped with this research.^21^ Qinghai and Gansu where the average life expectancy is low and the economic development is lagging. It is worth noting that the increase in the number of deaths, mortality and PAF of residents in Hunan Province caused by excessive intake of sugary beverages is high, suggesting that the province’s sugary beverage intake attributed death situation is relatively serious.

At the Ninth Global Health Promotion Conference held in 2016, China issued the ‘2030 Shanghai Declaration on Health Promotion in Sustainable Development’^22^ and the ‘Shanghai Consensus on Healthy Cities’,^23^ suggesting that the government should take action to promote the public health, use fiscal strategy and policy as a powerful tool to increase investment in health and well-being; strengthen legislation, regulation and taxation of unhealthy products. There are two health promotion projects generally carried out in China represented by China Healthy Lifestyle for All and the National Demonstration Areas for Comprehensive Prevention and Control of Chronic Diseases actively advocate the implementation of salt, oil and sugar reduction activities.^24^ At present, sugar reduction actions in China are mainly implemented by health education measures. Other measures such as compulsory food labeling, strengthening media publicity, guiding the market and enterprises to provide healthy and low-sugar beverages, and imposing sugar taxes are less practiced. WHO suggests that a 20% increase in taxes can effectively reduce the consumption of sugar-sweetened beverages.^25^ Based on international experiences, the taxation of sugar-sweetened beverages is mainly researched and practiced in developed countries such as European countries and the USA. Some states in the USA have begun to levy sugar taxes, and the UK, Denmark, and France have practiced and achieved certain effects on the issue of sugar taxes.^26–28^ Mexico, a developing country, is the first country in the world to implement a sugar tax, and the results have confirmed that 1 year after the sugar tax was imposed, the total consumption of sugar-sweetened beverages dropped by 12%, and the consumption of sugar-sweetened beverages by low-income people decreased is even more pronounced, which reached 17.4%.^29^^,^^30^ These countries have experienced the popularity of sugar-sweetened beverages and practiced sugar control strategies, and the experience and lessons learned should be used for reference by China.

This study describes for the first time the deaths of Chinese residents attributed to excessive intake of sugar-sweetened beverages, deaths in different provinces, and the burden of disease caused by excessive intake of sugar-sweetened beverages between 1990 and 2019. The results of the study found that in the past three decades, the number of deaths attributed to excessive intake of sugar-sweetened beverages among Chinese residents has nearly doubled. In 2019, the number of deaths attributed to excessive intake of sugar-sweetened beverages in China has exceeded 46 000. The proportion of deaths attributed to excessive intake of sugary beverages also increased by 35%. The burden of cardiovascular disease and diabetes deaths caused by excessive intake of sugar-sweetened beverages is particularly prominent. In particular, the disease burden of diabetes due to excessive intake of sugar-sweetened beverages has increased by nearly 90% in the past 30 years. The increase of YLDs for diabetes was 105%. Residents in Shandong, Henan, Hebei, Hunan and Guangdong provinces have a heavier burden of deaths attributed to excessive intake of sugar-sweetened beverages. Chongqing, Qinghai, Gansu, Xinjiang and Hunan provinces have seen significant increases in deaths. Henan, Qinghai and Gansu's sugar-sweetened beverages caused an increase in the standardized mortality rate, while Hunan, Gansu, Shandong, Henan and Yunnan sugary beverages caused the most significant increase in the proportion of deaths.

Based on the results of this study, we suggest that attention should be paid to the problem of excessive intake of sugar-sweetened beverages for Chinese population. In addition to the regular monitoring and investigation of sugar-sweetened beverage intake, comprehensive measures should be taken in China’s sugar control work. Recommended strategies and measures include the sugar composition of foods on food nutrition labels, the improvement of sugary food processing technology, the improvement of the market for sugary beverages through taxes or subsidies, the increase of the availability of water and healthy food, the creation of a healthy eating environment, and restrictions promotion of sugary beverages and advertising targeting children and adolescents, as well as public education and healthy diet consultation.

This study has several limitations. Although the current GBD calculation process has reassessed all existing evidence of the causal relationship between sugar-sweetened beverages and disease outcomes, and found sufficient evidence for the causal relationship between sugar-sweetened beverages, IHD and type 2 diabetes. Most of the research comes from high-income countries. Therefore, the results obtained may bias the estimation of the real situation in China. In order to reduce the bias, we encourage more original Chinese research on sugar-sweetened beverages to be published and included in the global burden of disease estimation framework.

## Funding

This study is supported by National Key R&D Program of China (No. 2018YFC1315304), Griffith University International Postgraduate Research Scholarship (GUIPRS) and Griffith University Postgraduate Research Scholarship.

*Conflicts of interest*: None declared.

## Ethical review

Ethics approval and consent from individuals were not required, as only aggregated non-identifiable data were used in this study.

Key points

- The number of deaths attributed to excessive intake of sugar-sweetened beverages among Chinese residents has nearly doubled from 1990 to 2019.
- The proportion of deaths caused by excessive consumption of carbon-containing beverages increased 35% from 1990 to 2019 in China.
- Excessive intake of sugar-sweetened beverages causes more serious disease burden on men in China.
